# Alien and Potentially Invasive Plants in Four Lagoons on the Island of Cozumel, Mexico

**DOI:** 10.3390/plants12091918

**Published:** 2023-05-08

**Authors:** Gonzalo Castillo-Campos, José G. García-Franco, M. Luisa Martínez, J. Jesús Pale-Pale

**Affiliations:** 1 Red de Biodiversidad y Sistemática, Instituto de Ecología A.C. (INECOL), Xalapa 91073, Mexico; gonzalo.castillo@inecol.mx (G.C.-C.); jesus.pale@inecol.mx (J.J.P.-P.); 2 Red de Ecología Funcional, Instituto de Ecología A.C. (INECOL), Xalapa 91073, Mexico; 3 Institute of Engineering, Universidad Nacional Autónoma de México, Mexico City 97302, Mexico

**Keywords:** aquatic vegetation, lagoon, protected natural area, control and management, biodiversity

## Abstract

The expansion of alien invasive species is a worldwide threat that affects most ecosystems. Islands and freshwater ecosystems are among the most vulnerable to species invasion, resulting in reduced biodiversity. In this study, we aimed to explore the floristic composition of the aquatic vegetation in four lagoons in southeastern Cozumel and assess the occurrence and abundance of alien and potentially invasive plants. We found a total of 43 aquatic or underwater herbaceous species that are subject to periodic flooding. Cluster analyses grouped the lagoons into two groups according to their floristic composition. The results demonstrate that alien and potentially invasive plants were dominant in 3 of the 4 lagoons, representing from 7 to 43% of the species. Six of these species were notably abundant, especially in three lagoons. Further, 2 species are considered among the 100 worst invasive species worldwide, although their abundance in Mexico remains relatively reduced. Five alien and potentially invasive species are terrestrial and grow on the shore of the lagoons, while one is aquatic. Urgent control and management actions are necessary. These should include (a) early detection and surveillance to determine if the alien species found behave as invasives; (b) understanding the relevance of invasive species; (c) preventing and intercepting; and (d) control and management. Habitat restoration, adequate legislation, collaboration between stakeholders, and raising awareness of the dangers of releasing or cultivating invasive species in the wild are also necessary.

## 1. Introduction

The expansion of alien invasive plants is occurring globally and seems like an unstoppable wave, affecting many, if not all, natural ecosystems [1]. Of these, freshwater ecosystems are among the most vulnerable to alien invasive species [2]. When invaded, the body of water and the surrounding land are particularly susceptible to drastic changes in community composition, structure, and biotic interactions [3]. Furthermore, the absence of physical barriers promotes the dispersal and expansion of alien invasive species within the water bodies, especially small lakes, and standing water. Additionally, human activities promote the accidental or deliberate introduction of alien invasive plants and animals into water bodies [4].

The integrity of freshwater ecosystems is highly relevant to humans and their well-being because they provide several relevant ecosystem services, such as drinking water, fisheries, pollution dilution, and recreation, among others [5]. Nevertheless, they are increasingly threatened by human activities such as pollution, eutrophication, and the colonization and expansion of alien invasive species. Furthermore, the impact of alien invasive species is most significant on island ecosystems, which harbor almost half of the world’s endangered biodiversity [6]. Thus, the occurrence of alien invasive species within island water bodies becomes especially relevant. Nevertheless, even though alien invasive species are acknowledged as drivers of biodiversity loss, studies on the pathways, drivers, mechanisms, and effects of alien invasive species are relatively scarce for island water bodies [2].

In Mexico, the island of Cozumel has been granted several protection measures because of its high biodiversity [7,8]. Despite such conservation efforts, several alien invasive species occur on the island. For instance, Castillo-Campos et al. [9] registered the first record of the naturalization of *Scaevola taccada* (Gaertn.) Roxb. on the beach and coastal dunes of Cozumel. Later, the same authors explored the effects of this Asian invasive species on plant community structure and composition [10]. Similarly, García-Arroyo et al. [11] found that the Eurasian collared-dove (*Streptopelia decaocto* (Frivaldszky, 1838)), one of the most successive birds worldwide, was very abundant in the urban areas of the island. Additional alien invasive species reported in Cozumel include the lionfish (*Pterois volitans* (Linnaeus, 1957)) [12]; a tarantula (*Brachypelma vagans* (Ausserer, 1875)) [13]; and the boa (*Boa imperator* (Daudin, 1803)) [14]. The island of Cozumel contains several water bodies, but these have not been studied, so it is unknown if alien invasive species have also colonized these. Because of the relative abundance of alien invasive species on the island, and the vulnerability of water bodies to invasion, our premise was that it is very likely that water bodies would also be affected by alien invasives.

In this context, first, we aimed to study the floristic composition of four lagoons located in southeastern Cozumel and assess the occurrence, frequency, and abundance of alien and potentially invasive plants. Second, we report (a) the geographic origin of these alien species, (b) their geographic distribution, and (c) the potential mechanisms of plant invasions on the island inferred from the use of these plants by humans. With this information, we analyze future challenges and make recommendations for preventing and controlling the expansion of alien and potentially invasive species in the lagoons of Cozumel.

## 2. Results

### 2.1. Floristic Composition and Sampling Effort

The lagoons sampled are surrounded mainly by or immersed in a low tropical deciduous forest [15] and, to a lesser extent, mangroves (*Conocarpus erectus*) or flooded forests characterized by *Annona glabra* L., *Cresentia alata* Kunth, *Erythroxylum confusum* Britton, and *Bonellia albiflora* (Lundell) B. Stahl & Källersjö.

In the inventory of the plots sampled in the 4 lagoons, we registered 43 species (Table 1) of aquatic or underwater flora belonging to 24 families (27 species in lagoon 1; 15 species in lagoon 2; 22 species in lagoon 3; and 7 species in lagoon 4). These species are subject to periodic flooding and are primarily herbaceous. The occurrence of one alga (*Chara haitensis*) is noteworthy because of its relatively high abundance. This species was the only alga registered in our sampled plots.

Most species belonged to the herbaceous flora, characterized by *Bacopa monnieri* (L.) Wettst., *Ceratophyllum demersum* L., *Echinodorus berteroi* (Spreng.) Fasset, *Eleocharis elegans* (Kunth) Roem. & Schult., *E. geniculata* (L.) Roem. & Schult., *Marsilea vestita* Hook. & Grev., *Nymphaea ampla* (Salisb.) DC., *Paspalidium geminatum* (Forssk.) Stapf, *Paspalum vaginatum* Sw., and *Stachytarpheta angustifolia* (Mill.) Vahl. These species form dense mats that almost completely cover the humid zone surrounding the water bodies of the four lagoons. Two alien and potentially invasive plants, *Paspalum vaginatum* and *Paspalidium geminatum,* were among the most abundant and dominant species.

Notably, two of the species found are native to North America (Mexico inclusive) but are considered aggressive alien invasive plants elsewhere. For instance, *Ceratophyllum demersum* is distributed worldwide because of its general use in aquariums and ponds. It is a submerged aquatic plant that may form dense monospecific beds, resulting in the exclusion of other plant species [16]. This species is among the 100 worst invasive species worldwide. *Bacopa monnieri* is also considered native to the Americas, Asia, and Africa but is invasive in Japan, Singapore, Spain, Portugal, and the Cayman Islands. Like *Ceratophyllum demersum*, *Bacopa monnieri* grows in aquatic habitats and is an ornamental plant in aquariums and ponds. It has deleterious effects on native plants and animals, water quality, water flow, and sedimentation [17].

**Figure 1:**
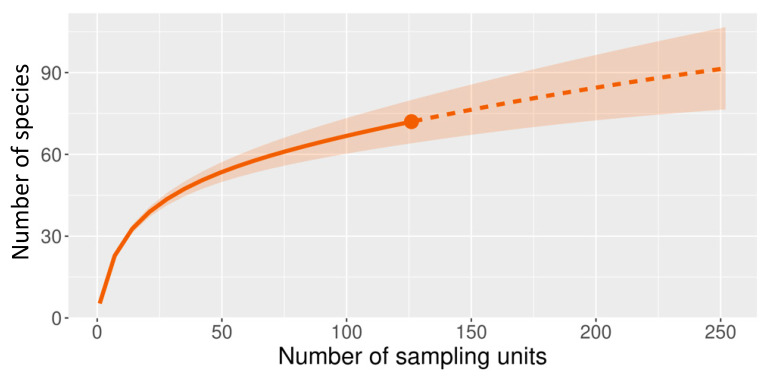
Rarefaction curves for species richness (q0) calculated with incidence values of the plant species found in four lagoons on the island of Cozumel in Quintana Roo, Mexico.

The rarefaction analysis based on the number of sampling units shows that species richness did not reach the expected asymptotic value, which indicates the necessity of increasing the number of sampled units. Nevertheless, the estimated cover sample values (CS 0.9642) reveal that the sampling effort was sufficient (72) (Figure 1).

### 2.2. Floristic Analysis

**Figure 2:**
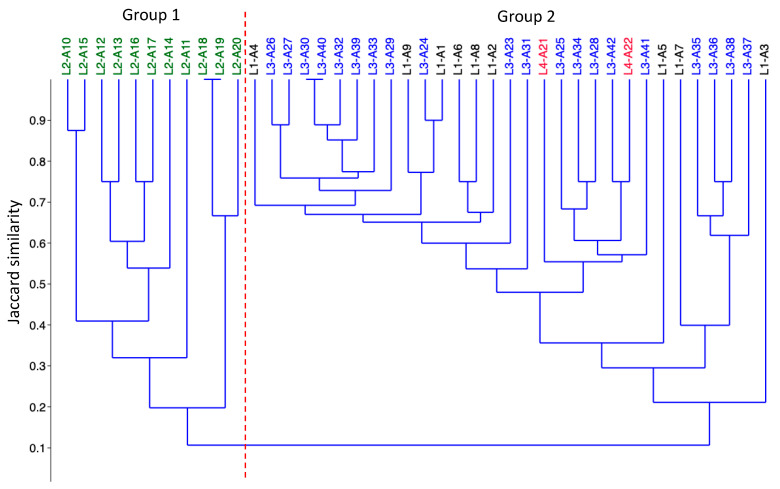
Cluster analysis based on Jaccard index shows floristic similarity between the sampled plots. Dashed red lines differentiate vegetation groups. Color labels in each group show the plots sampled in each lagoon: Black = Lake 1, Green = Lake 2, Blue = Lake 3, and Red = Lake 4.

The cluster analysis distinguished two groups of plant communities (Figure 2). In these plant communities’ groups, the species richness was composed of 15 species in group 1 and 35 species in group 2. The vegetation groups can be distinguished by the species turnover between them and the presence and dominance of some species.

Group 1 contains all the samples from lagoon 2, the one furthest away from the other lagoons (12 km apart, see methods). This group was mainly characterized by five species *Eleocharis elegans*, *Eragrotis prolifera*, *Bocopa monnieri*, *Paspalum virgatum*, and *Nymphoides indica* (Table 1).

Group 2 contained all sampled plots from lagoons 1, 3, and 4, possibly owing to the smaller distance between them (250–290 m, see methods) (Figure 2). The lagoons shared many species, but the principal species in group 2 were *Echinodorus berteroi*, *Eleocharis geniculata*, *Marsilea vestita*, *Paspalidium geminatum*, and *Paspalum vaginatum*.

It is important to mention that we recorded 6 alien and potentially invasive species (Table 1), and 1–4 of these occurred in each lagoon. In lagoon 1, *Cynodon dactylon, Dactyloctenium aegyptium*, *Paspalidium geminatum*, and *Paspalum vaginatum* were present. In lagoon 2, only *D. aegyptium* was recorded. In lagoon 3, *Nymphae rubra*, *P. geminatum*, *P. vaginatum*, and *Portulaca oleracea* were observed. Finally, in lagoon 4, *D. aegyptium*, *Paspalidium geminatum*, and *Paspalum vaginatum* were recorded. Three grass species (*Paspalum vaginatum*, *Paspalidium geminatum*, and *Dactyloctenium aegyptium*) grow in all the lagoons studied. The most abundant alien and potentially invasive species was *Paspalum vaginatum*, while the least frequent were *Cynodon dactylon*, *Nymphaea rubra*, and *Portulaca oleracea*.

### 2.3. Relative Frequency and Abundance of Alien and Potentially Invasive Plants

**Figure 3:**
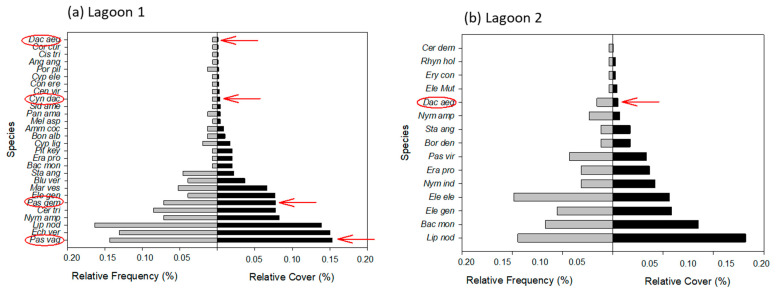

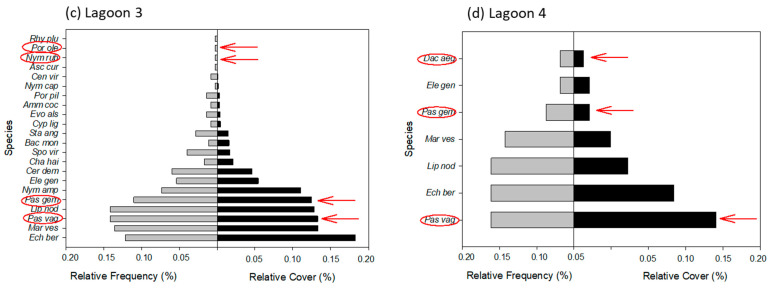
Relative frequency and cover of the six alien and potentially invasive species compared with the native plants observed in four lagoons on the island of Cozumel, Mexico. Red arrows and circles indicate the potentially invasive species. The acronyms are related to the species in Table 1.

The relative frequency and abundance of the alien plants, potentially invasive in Cozumel, varied between lagoons and between species (Figure 3). In all lagoons, except for lagoon 2, the relative cover of at least two species (*Paspalum vaginatum* and *Paspalidium geminatum*) was amongst the highest. In contrast, only 1 alien and potentially invasive species (*Dactyloctenium aegyptium*) was observed in lagoon 2, with a reduced plant cover. The results demonstrate that alien and potentially invasive plants were dominant in three of the four lagoons. For instance, these species represent 14 and 18% of the flora in lagoons 1 and 3, respectively. The extreme percentages occurred in lagoon 2, with only 1 alien and potentially invasive species representing 7% of the flora, and species-poor lagoon 4, with 3 alien and potentially invasive species representing 43% of the flora.

### 2.4. Geographic Distribution of the Alien and Potentially Invasive Plants

**Figure 4:**
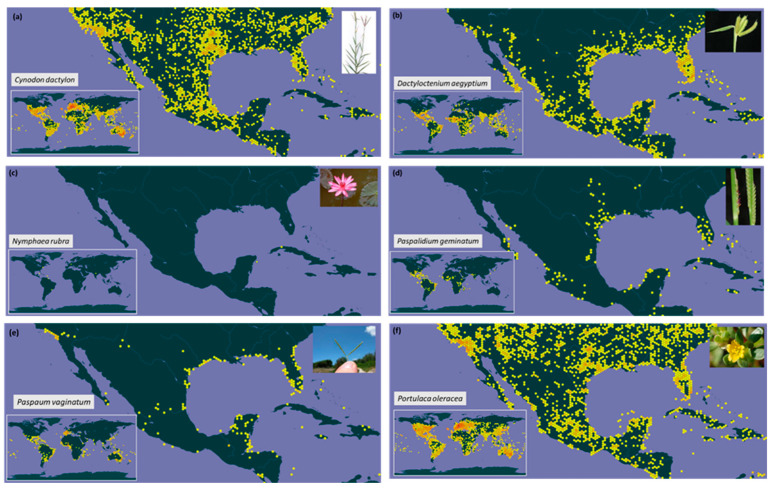
Geographic distribution of the six alien and potentially invasive species found in four lagoons on the island of Cozumel, Mexico, based on the Global Biodiversity Information Facility (GBIF) database (https://www.gbif.org/, accessed on 13 February 2023). Inserted subfigures (**a**–**f**) show maps with the global distribution of each species. Each point indicates a record of the species in a locality. The orange-reddish dots represent a larger number of records at that location. Inserted photos: under creative commons license.

The worldwide and Mexican distributions of alien plants that are potentially invasive in Cozumel are variable (Figure 4). For instance, *Cynodon dactylon, Dactyloctenium aegyptium*, and *Portulaca oleracea*, whose centers of origin are in Africa, the Old World, and Asia, respectively, are widely distributed worldwide and in Mexico (Table 2). *Cynodon dactylon* is one of the 100 worst invasive species in the world. The local distribution of *Paspalum geminatum* and *Paspalidium vaginatum* (native from the USA) in Mexico is reduced. Still, *Paspalum vaginatum* is another invasive plant among the world’s 100 worst invasive species (Figure 4, Table 2). The least broadly spread alien and potentially invasive species was *Nymphaea rubra*, native to Asia, recently reported in Cozumel [9]. It is important to state that, so far, we do not have evidence to determine if these alien species can already be considered invasives in the lagoons, although some of them were already very abundant (*Paspalum vaginatum* and *Paspalidium geminatum*). Rather, they are considered potentially invasive because they have been reported as invasives in Mexico. Nevertheless, field data is necessary to determine if the species are indeed acting as invasives.

## 3. Discussion

In this study, we aim to describe the floristic composition of four lagoons located on the southeastern coast of the island of Cozumel and assess the occurrence and abundance of alien and potentially invasive plant species. Our results reveal a total of 43 species of aquatic or underwater flora, which are mostly herbaceous plants, subject to periodic flooding. Six alien and potentially invasive species were notably abundant, especially in three lagoons. Further, 2 are considered among the 100 worst invasive species worldwide, although their abundance in Mexico remains relatively reduced. Five alien and potentially invasive species are terrestrial and grow on the shore of the lagoons, while one is aquatic.

### 3.1. Alien Potentially Invasive Species

A relatively large number of the species found in the 4 lagoons (13.9%) were alien, and considered potentially invasive, since they have been reported as invasives elsewhere in Mexico and in different types of ecosystems and environments. For example, *Cynodon dactylon* was considered potentially invasive in the mountain ranges along the Mexican Pacific coast [24] and in northwestern Mexico (Baja California) [25] after its introduction for grazing.

*Dactyloctenium aegiptium* was also observed on an island located in northwestern Mexico [26] and on coastal dunes in southeastern Mexico [27] and is considered potentially invasive. However, Zaldívar-Cruz et al. [28] did not consider this grass invasive, although it was observed growing in association with the invasive tree *Casuarina equisetifolia* along the coasts of Cozumel [28]. *C. dactylon* and *D. aegiptium* have also been mentioned as alien invasive plants in the riparian zones of the Sonoran Desert [29].

Other species are included in lists of alien invasive species in Mexico, but studies on them for Mexican populations are almost non-existent. For instance, *Nymphaea rubra* has only been recently recorded in other lagoons in Cozumel and was mentioned as possibly being invasive [10]. *Paspalidium geminatum* is mentioned as being invasive in Mexico by the National Commission of Biodiversity (CONABIO) [21], and the Global Invasive species database [22] acknowledges *Paspalum vaginatum* as invasive in Mexico. *Portulaca oleracea* is also referred to as invasive in Mexico by international databases [23]. However, we did not find scientific studies on these species in Mexico. The above highlights the urgent need for further in-depth studies on these potentially invasive species of Mexico.

### 3.2. The Routes and Possible Invasion Mechanisms and Impacts of Alien and Potentially Invasive Species in the Lagoons of the Island of Cozumel

The successful colonization and establishment of alien invasive plants depend on different factors: first, invasion pathways; second, driving factors that provide invasion opportunity windows; third, ecological mechanisms that promote a successful invasion. Finally, combining these factors determines the effects of invasive species in natural communities [2]. Next, we describe the possible invasion pathways, invasion drivers, invasion mechanisms, and invasion effects of the alien potentially invasive species found in the lagoons.

**Invasion pathways**—In aquatic systems, ornamental trade frequently underlies escape from captivity and deliberate release [2]. These actions occur primarily because of a general lack of knowledge regarding the potential danger of released invasive species. In our case, the only truly aquatic potentially invasive plant was *Nymphae rubra* which is an ornamental plant in ponds. The introduction of this plant to the island is possibly the result of its astonishingly beautiful flowers. Bird dispersal probably contributed to the expansion of this species [8].

The other alien and potentially invasive species observed in our study sites are typically associated with grasslands for cattle ranching. It is likely that they were introduced for cattle and then escaped from these disturbed sites. Some evidence for this is the observed cattle tracks (prints and droppings) in all our study sites and a few individuals of wild boar (*Pecari tajacu nanus*) grazing on the shores of lagoon 3.

**Invasion drivers**—Different environmental conditions are relevant for species invasion. Nutrient contents, floristic structure, vegetation density, climate, and hydrology oftentimes play a decisive role in the colonization, establishment, and invasion of alien species [24,25]. Because the lagoons we studied are on an island, the most likely drivers of colonization are human activities (cattle ranching) and decisions (ornamental plants).

**Invasion mechanisms**—In general, invasive plants show effective dispersal and spread mechanisms [30,31,32]. The seeds of the alien and potentially invasive species found in the four lagoons are tiny, and their dispersion means are associated with wind and the movement of birds because the seeds can become attached to their feathers or legs. Furthermore, because the lagoons are relatively close, dispersion between them is very likely. Additionally, clonal growth of the six species has probably promoted their expansion on the shores of the lagoons [33,34].

**Invasion effects**— If the observed alien and potentially invasive species expand their cover in the lagoons, it is likely that they will behave as invasives and have a relevant effect in the lagoons. Thus, even though we did not explore the long-term impact on community composition, structure, and dynamics, the high frequency of alien and potentially invasive plants is likely to significantly affect the functional integrity of these lagoons. Further studies are necessary in this regard.

### 3.3. Future Challenges and Recommendations

The control and management of invasive species require a program that includes a combination of actions [35]: (a) early detection and surveillance of alien and potentially invasive species to determine if they become invasive or not; (b) understanding the relevance of invasive species from a scientific and a socioeconomic point of view; (c) preventing and intercepting alien and potentially invasive species; and (d) control and management of invasive species. In Cozumel, we are only at the beginning with early detection data, and from this, we need to move forward. For instance, it is necessary to determine if these alien species become invasive (or begin to exhibit invasive behavior) as soon as possible. Then, it is necessary to implement, for example, the actions recommended by [35]. Supplementary management actions to control alien invasive plants should include habitat restoration, adequate legislation, collaboration between stakeholders, and raising awareness of the dangers of releasing or cultivating invasive species in the wild [4].

## 4. Materials and Methods

### 4.1. Study Site

The study took place on the island of Cozumel, located in the Mexican Caribbean area, 17 km off the eastern coast of the Yucatan Peninsula, Mexico (20°3″ N and 87°3″ W; Figure 1). The climate is warm sub-humid with a mean annual temperature of 26–27 °C and annual rainfall between 800 and 1500 mm. March and April are the driest months, with the highest rainfall in September [36]. Frequently, tropical storms or hurricanes hit the island during the summer, making landfall on the Caribbean part of the island. Human settlements and infrastructure are located on the other side of the island and are better protected from these storms.

The vegetation on the island grows on a highly permeable limestone and sandy substrate. Different ecosystems grow here: sub-deciduous tropical forests, mangrove swamps, wetlands, and coastal dune vegetation [9,10,37,38,39].

### 4.2. Vegetation Sampling

**Figure 5:**
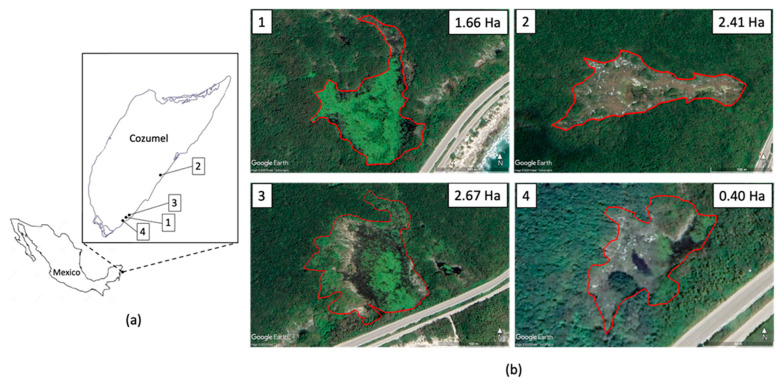
(**a**) Location of the island of Cozumel in the state of Quintana Roo, southeastern Mexico. The numbers indicate the lagoons studied. (**b**) Studied lagoons. In each lagoon, the red line shows the surface that is potentially flooded during heavy rainfalls. The flooded surface is shown in the inserted values. Images from Google Earth.

The vegetation was recorded in four lagoons located on the southeastern side of the island of Cozumel (Caribbean side) (Figure 5). The distances between the 3 lagoons varied between 250 and 290 m (Lagoons 1, 3, 4), and the fourth (Laguna 2) was 11 km from the first. The lagoons studied were selected on the basis of their accessibility and the presence of the water mirror at the time of the study. The lagoons had different dimensions of the body of water present during sampling and the potentially floodable area (Figure 5).

Vegetation sampling was carried out through transects oriented perpendicular to the lagoons, beginning in the water mirror where the floating leaf species grow and ending inland, where the last traces of the maximum rainy season flooding were observed. The length of the transects varied depending on the width of the margin of the water bodies, and the transects were located at different points in each lagoon.

In each transect, we established 1–3 sampling squares that were 5 m wide × 20 m long (100 m^2^) and separated by 25 m. The first sampled plot was always in the water 10–15 m from the shore, where the water reached a depth of 30–35 cm, and the species with floating leaves occurred. The resulting plots included plants that withstand flooding during the rainy season but are on the land when the water level drops during the dry season. In this way, we included all the species present in the different flooding conditions.

We sampled all woody species in the 100 m^2^ plots. Then, we recorded herbaceous species in three 2 × 2 m subplots randomly placed within the 100 m^2^ ones. In each plot and subplot, we listed all the species and visually estimated the percent cover of each one (0–100%). Because of this method, and because plant covers overlapped, the sum of the plant cover of all the species was greater than 100% in the most densely vegetated plots. We sampled 42 plots (100 m^2^) covering a total area of 4200 m^2^. The total number of plots sampled in each lagoon was as follows: lagoon 1 = 9 plots; lagoon 2 = 11; lagoon 3 = 20; and lagoon 4 = 2. We sampled 126 subplots (4 m^2^), corresponding to 504 m^2^. In this case, the number of subplots per lagoon was as follows: lagoon 1 = 27 subplots (109 m^2^); lagoon 2 = 33 (132 m^2^); lagoon 3 = 60 (240 m^2^); and lagoon 4 = 6 (24 m^2^). It is important to note that the number of sampled transects, plots, and subplots in each lagoon depended on the size of the water body present at the time of the study (October 2021) and the potentially floodable area at the time of the highest content of water.

Because the Island of Cozumel is a Biosphere Reserve, collecting specimens for scientific collections is not allowed, so the plant species were identified during sampling. When a species could not be determined in situ, we used high-quality photographs taken with a Canon SX270 HS 20x zoom lens for subsequent identification. We employed Family taxonomic keys and performed comparisons with herbarium specimens (XAL). The scientific names of the registered species were corroborated through the electronic databases (Tropicos.org) [40] and online herbaria data (MEXU, XAL), as well as by consulting the floristic lists published for the lagoons and wetlands of the Yucatan Peninsula [36,37,38,39,41].

### 4.3. Data Analyses

#### Occurrence and Abundance

Initially, we analyzed the sampling efficiency with a species accumulation curve using the online program iNEXT [42]. Subsequently, we explored the floristic similarity between the sample units using multivariate Hierarchical cluster analysis (UPGMA) and calculated the Jaccard similarity index with PAST 4.11 [43]. This index relates the presence or absence of the species. Using this analysis, we identified floristic affinities between the plots and lagoons. We expected that the sampled plots of each lagoon would be grouped according to their floristic affinities, that is, that four groups would be formed, one for each lagoon.

On the other hand, we obtained the relative importance value (RIV) of each species using Equation (1):

RIV = (RF + RA/2) × 100(1)

where RF is the relative frequency, and RA is the relative coverage (as abundance value). This way, we identified the most important species in the community and each lagoon.

### 4.4. Distribution Worldwide and in Mexico of the Alien and Potentially Invasive Species

We used previous studies and public databases (Global Invasive Species Database) [16] and CONABIO [44] to determine the status (native or invasive) of the species found in the four lagoons. Then, we designated those species considered invasive in Mexico as potentially invasive in the lagoons of Cozumel, even though we did not collect evidence to confirm their invasiveness (see Table 2). Then, the distributions worldwide and in Mexico of the alien invasive species found in the four lagoons were mapped on the basis of the information from the Global Biodiversity Information Facility [45].

## 5. Conclusions

This study shows that six alien species are potentially invasive, and of these, three were notably abundant in the lagoons we studied on the island of Cozumel. Although additional studies are still necessary to determine if these alien species are indeed becoming invasives, it is also necessary to understand invasion routes, pathways, mechanisms, and impacts. Using such information, different management actions can be implemented, and these include (a) early detection and surveillance; (b) understanding the relevance of invasive species from a scientific and a socioeconomic point of view; (c) preventing and intercepting invasive species; and (d) control and management of invasive species. Additionally, habitat restoration, adequate legislation, collaboration between stakeholders, and raising awareness of the dangers of releasing or cultivating invasive species in the wild are also necessary.

## Figures and Tables

**Table 1:** Aquatic and subaquatic species observed in four lagoons located in southeastern Cozumel, Mexico. Acronyms of each species are shown. Asterisks indicate the alien and potentially invasive plants found. The relative importance values are shown for each species and for each lagoon; bold letters indicate the largest values in each lagoon.

Family	Species	Acronyms	L1	L2	L3	L4
Alismataceae	*Echinodorus berteroi* (Spreng.) Fasset	Ech ber	**16.6**	0	**14.4**	**21.3**
Amaranthaceae	*Blutaparon vermiculare* (L.) Mears	Blu ver	2.9	0	0	0
Apocynaceae	*Asclepias curassavica* L.	Asc cur	0	0	0.1	0
Asteraceae	*Melanthera aspera* (Jacq.) Steud. ex Small	Mel asp	0.7	0	0	0
Ceratophyllaceae	*Ceratophyllum demersum* L.	Cer dem	**11.7**	0.5	7.7	0
Characeae	*Chara haitensis* D. E. Berthold	Cha hai	0	0	2.0	0
Combretaceae	*Conocarpus erectus* L.	Con ere	0.5	0	0	0
Convolvulaceae	*Evolvulus alsinoides* (L.) L.	Evo als	0	0	0.8	0
Cordiaceae	*Cordia curassavica* (Jacq.) Roem. & Schult.	Cor cur	0.3	0	0	0
Cyperaceae	*Cyperus elegans* L.	Cyp ele	0.5	0	0	0
	*Cyperus ligularis* L.	Cyp lig	1.4	0	0.5	0
	*Eleocharis elegans* (Kunth) Roem. & Schult.	Ele ele	0	**25.3**	0	0
	*Eleocharis geniculata* (L.) Roem. & Schult.	Ele gen	4.5	**10.0**	5.4	2.1
	*Eleocharis mutata* (L.) Roem. & Schult.	Ele mut	0	1.1	0	0
	*Rhynchospora holoschoenoides* (Rich.) Herter	Rhy hol	0	0.4	0	0
	*Rhynchospora plumosa* Elliott	Rhy plu	0	0	0.1	0
Erythroxylaceae	*Erythroxylum confusum* Britton	Ery conf	0	0.4	0	0
Fabaceae	*Centrosema virginianum* (L.) Benth.	Cen vir	0.3	0	0.4	0
	*Pithecellobium keyense* Britton	Pit key	0.5	0	0	0
Lythraceae	*Ammannia coccinea* Rottb.	Amm coc	1.1	0	0.5	0
Marsileaceae	*Marsilea vestita* Hook.& Grev.	Mar ves	4.9	0	**18.4**	**23.5**
Menyanthaceae	*Nymphoides indica* (L.) Kuntze	Nym ind	0	5.4	0	0
Nymphaeaceae	*Nymphaea ampla* (Salisb.) DC.	Nym amp	6.7	3.0	8.2	0
	*Nymphaea capensis* Thunb.	Nym cap	0	0	0.3	0
	*Nymphaea rubra* Roxb. ex Salisb. *	Nym rub	0	0	0.5	0
Plantaginaceae	*Angelonia angustifolia* Benth.	Ang ang	0.3	0	0	0
	*Bacopa monnieri* (L.) Wettst.	Bac mon	0.5	**14.5**	0.7	0
Poaceae	*Cynodon dactylon* (L.) Pers. *	Cyn dac	0.6	0	0	0
	*Dactyloctenium aegyptium* (L.) Willd. *	Dac aeg	0.4	1.6	0	2.0
	*Eragrostis prolifera* (Sw.) Steud.	Era pro	0.5	4.3	0	0
	*Panicum amarum* Elliott	Pan ama	1.6	7.4	0	0
	*Paspalidium geminatum* (Forssk.) Stapf *	Pas gem	5.7	0	8.8	4
	*Paspalum vaginatum* Sw. *	Pas vag	**13.7**	0	**9.5**	**20.2**
	*Paspalum virgatum* L.	Pas vir	0	**12.2**	0	0
	*Sporobolus virginicus* (L.) Kunth	Spo vir	0	0	2.6	0
Portulacaceae	*Portulaca oleracea* L. *	Por ole	0	0	0.1	0
	*Portulaca pilosa* L.	Por pil	0.7	0	0.8	0
Primulaceae	*Bonellia albiflora* (Lundell) B. Ståhl & Källersjö	Bon alb	0.7	0	0	0
Rubiaceae	*Borreria densiflora* DC.	Bor den	0	1.4	0	0
Sapotaceae	*Sideroxylon americanum* (Mill.) T.D. Penn.	Sid ame	0.4	0	0	0
Verbenaceae	*Lippia nodiflora* (L.) Michx.	Lip nod	**18.5**	**18.54**	**16.2**	**26.9**
	*Stachytarpheta angustifolia* (Mill.) Vahl	Sta ang	3.5	1.4	1.9	0
Vitaceae	*Cissus trifoliata* (L.) L.	Cis tri	0.4	0	0	0

**Table 2:** Attributes of the six alien and potentially invasive plants found in four lagoons on the island of Cozumel. The acronyms of each species are explained in Table 1.

Species	Habitat	Growth Form	Use	Status in Mexico	Origin	UICN Status	Reference
*Cyn dac*	Terrestrial	Herb	Cattle	Invasive	Africa	100 worst	[18]
*Dac aeg*	Terrestrial	Herb	Cattle	Invasive	Old World		[19]
*Nym rub*	Freshwater	Herb	Ornamental	Invasive	Asia		[20]
*Pas gem*	Terrestrial	Herb	Unknown	Introduced	USA		[21]
*Pas vag*	Terrestrial/Freshwater	Herb	Cattle	Invasive	USA	100 worst	[22]
*Por ole*	Terrestrial	Herb	Food	Old exotic (invasive)	Asia		[23]

## Data Availability

Data are available upon request.
